# Supplementary material for: Lower Free Thyroxine Levels Are Associated with Diabetic Kidney Disease in Males with Type 2 Diabetes Mellitus: An Observational Cross-Sectional Study
Source: Biomedicines. 2024 Oct 17;12(10):2370. doi: 10.3390/biomedicines12102370 (PMC11504375; doi:10.3390/biomedicines12102370)
Supplement: Supplementary file 1 [file biomedicines-12-02370-s001.zip › biomedicines-3180322-supplementary.pdf]

Table S1. The correlations of thyroid function and diabetic kidney disease-related parameters.

		TSH	T4	T3	FT4	FT3	TGAB	TMAB	TPOAB
T2DM duration	r	0.06319732	-0.042964263	-0.04741523	-0.101600596	-0.093288277	0.000267484	0.023242821	0.017173276
	p	2.51E-05	0.004608397	0.001765817	1.84E-11	7.01E-10	0.985933534	0.125471393	0.280804081
HbA1c	r	-0.113604269	-0.112107239	-0.198950969	-0.043850805	-0.143830869	0.002167966	-0.004954727	-0.007223815
	p	7.96E-13	2.76E-12	8.50E-36	0.006344622	2.36E-19	0.89281974	0.758133093	0.668198323
Glycated albumin	r	-0.071997639	-0.115653972	-0.220264482	-0.071722767	-0.171412927	-0.000664853	-0.003806545	-0.019086891
	p	2.16E-05	1.39E-11	1.41E-38	2.83E-05	7.70E-24	0.969110048	0.824536807	0.287751557
UA	r	0.038503432	-0.028338924	0.031116689	0.018057721	0.020014807	-0.042032015	-0.042619137	-0.048280862
	p	0.012194527	0.068103185	0.045153108	0.244759774	0.197307773	0.006800246	0.006063812	0.003071593
eGFR	r	-0.154613281	0.067628067	0.165934315	0.137100719	0.209795816	-0.026382033	-0.033409502	-0.028625714
	p	2.59E-18	0.000175634	2.06E-20	2.06E-14	5.11E-32	0.143703363	0.064053492	0.131383214
FBG	r	-0.09284286	-0.047432232	-0.135018064	-0.052989299	-0.081485021	-0.019540883	-0.018681953	-0.030318678
	p	1.30E-09	0.002207274	2.17E-18	0.000620778	1.38E-07	0.20752739	0.228210046	0.062550229
Cys-C	r	0.00049776	-0.033718225	-0.106710663	-0.074052536	-0.173976877	0.048363016	0.037670176	0.017699321
	p	0.974193981	0.030165216	6.04E-12	1.84E-06	1.71E-29	0.001868105	0.015427876	0.278678838
Blood creatinine	r	0.063914206	-0.105086683	-0.114276068	-0.084374356	-0.130449549	-0.026715429	-0.036340284	-0.055206294
	p	3.23E-05	1.28E-11	1.75E-13	5.48E-08	3.64E-17	0.086004084	0.019503836	0.000725184
Blood urea nitrogen	r	0.018966132	-0.085084486	-0.12198366	-0.082027998	-0.132564649	-0.03279056	-0.028419376	-0.011185446
	p	0.217854699	4.33E-08	3.62E-15	1.27E-07	1.11E-17	0.035076704	0.067788444	0.493784487
Urinary creatinine	r	-0.065789211	0.045287255	0.199786459	0.118409493	0.232894494	-0.058363867	-0.065138452	-0.04260421
	p	7.72E-05	0.00710508	3.99E-33	1.66E-12	1.00E-44	0.000519815	0.000107079	0.01659921
UACR	r	0.013991797	-0.037860436	-0.169569058	-0.141644587	-0.162219902	-0.027001545	-0.010704447	-0.012924032
	p	0.401324899	0.024545248	3.66E-24	2.84E-17	3.13E-22	0.108867002	0.525092921	0.467895318
24-hour microalbumin	r	0.024061577	-0.030960924	-0.109097656	-0.109298535	-0.101183262	-0.034171044	-0.021629723	-0.042190702

	p	0.123497498	0.04991517	4.27E-12	3.69E-12	1.28E-10	0.030457171	0.17081283	0.011003906
24hU-TP	r	-0.019935206	-0.067316457	-0.125038262	-0.133981855	-0.166900837	0.028439057	0.014720412	-0.037593937
	p	0.201330562	1.93E-05	1.74E-15	1.31E-17	1.43E-26	0.071327377	0.350655938	0.023338727
24-hour urine output	r	-0.037962377	-0.072036947	-0.071515941	-0.026527333	-0.062570888	-0.004899494	-0.014226352	-0.007294632
	p	0.015147388	5.05E-06	5.91E-06	0.092871438	7.28E-05	0.756613294	0.368137439	0.660669689
Microalbumin	r	0.031744547	0.015323829	-0.052336632	-0.133055817	-0.025787786	0.075095617	0.145753256	0.001397978
	p	0.042756957	0.333373478	0.0009467	2.94E-17	0.102991592	2.07E-06	2.25E-20	0.933101939

TSH, thyrotropin; T4, thyroxine; T3, triiodothyronine; FT4, free thyroxine; FT3, free triiodothyronine; TMAB, thyroid microsomal antibody; TPOAB, thyroid peroxidase antibody; TGAB, thyroglobulin antibody; eGFR, estimated glomerular filtration rate; HbA1c, glycated hemoglobin; FBG, fasting blood glucose; Cys-C, cystine C; UACR, urinary albumin-to-creatinine ratio; 24hU-TP, 24-hour urine protein. UA, uric acid.

Table S2. Association between thyroid hormones and diabetic kidney disease stages

	eGFR-based staging			UACR-based staging		
	Coefficients	OR (95% CI)	P value	Coefficients	OR (95% CI)	P value
T3	-1.550	0.213 (0.155-0.291)	**<0.001**	-1.040	0.354 (0.282-0.443)	**<0.001**
T4	-0.094	0.911 (0.873-0.949)	**<0.001**	-0.057	0.945 (0.916-0.973)	**<0.001**
FT3	-0.512	0.599 (0.549-0.653)	**<0.001**	-0.312	0.732 (0.687-0.778)	**<0.001**
FT4	-0.108	0.897 (0.873-0.922)	**<0.001**	-0.076	0.927 (0.908-0.945)	**<0.001**
TSH	0.026	1.027 (1.016-1.038)	**<0.001**	0.005	1.005 (0.995-1.015)	0.319

Ordered logistic regression to explore the association between thyroid hormones and diabetic kidney disease (DKD) stages. DKD were classified into 1-5 stages by eGFR or 1-3 stages by ACR via KDIGO consensus. eGFR, estimated glomerular filtration rate; UACR, urinary albumin-to-creatinine ratio; OR, odds ratio; CI, confidence interval; T3, triiodothyronine; T4, thyroxine; FT3, free triiodothyronine; FT4, free thyroxine; TSH, thyrotropin. Bold indicates a significant P value < 0.05.
